# Housing type and myopia: the mediating role of parental myopia

**DOI:** 10.1186/s12886-016-0324-z

**Published:** 2016-08-31

**Authors:** Xiaoyan Wu, Guopeng Gao, Juxiang Jin, Wenjuan Hua, Liming Tao, Shaojun Xu, Fangbiao Tao

**Affiliations:** 1 Department of Maternal, Child & Adolescent Health, School of Public Health, Anhui Medical University, 81 Meishan Road, Shushan District, Hefei, Anhui Province 230032 China; 2 Department of Epidemiology and Biostatistics, School of Public Health, Anhui Medical University, Hefei, 230032 China; 3 Department of Ophthalmology, the Second Affiliated Hospital of Anhui Medical University, Hefei, 230601 China

**Keywords:** Myopia, School-aged children, Housing type

## Abstract

**Background:**

Myopia has become a significant global public health concern, and is highly prevalent worldwide especially in Asian countries. It is associated with genetic factors as well as socioeconomic status; however, the underlying cause for school myopia has not been established. This study evaluates the impact of living environment on school myopia in Chinese school-aged children.

**Methods:**

A large cross-sectional sample of area- and ethnicity-matched school children; a total of 43, 771 children from 12 cities participated in this study. The presence of myopia was self-reported and potential risk factors were determined by questionnaires.

**Results:**

The self-reported prevalence of myopia in Chinese children was 31.8 % (*n* = 13, 928). In multiple logistic regression analysis, higer risk of myopia among school-aged children was significantly positively associated with both parental myopia (*OR* = 3.57; 95 % *CI*: 3.26–3.90), living in 1–3 floor (*OR* = 1.28; 95 % *CI*: 2.57–3.15), 4–6 floor (*OR* = 1.84; 95 % *CI*: 1.73–1.95) and 7 floor or more (*OR* = 2.02; 95 % *CI*: 1.88–2.16). Particularly, housing type was independently associated with myopia after stratified by parental myopia. An increasing prevalence of myopia was found with increasing floor of housing type in each outdoor time group.

**Conclusions:**

Housing type was independently associated with myopia, after stratified by parental myopia. Flat room, lower living floor and more outdoor time may be protective factors for myopia among school-aged children in mainland China.

**Electronic supplementary material:**

The online version of this article (doi:10.1186/s12886-016-0324-z) contains supplementary material, which is available to authorized users.

## Background

Myopia, which is often regarded as correctable cause of visual impairment, has become a significant global public health concern [1]. In economically developed societies, myopia develops during childhood for the most part, particularly during the school years [2]. Epidemiological studies have indicated a high prevalence of myopia worldwide, especially in Asian countries [3]. In urban areas in east and southeast Asian countries, 80 % to 90 % of children completing high school are myopic, whereas 10 % to 20 % are high myopia [4]. Moreover, myopia affects 29 % of primary school children in Singapore [5]. Currently, children are developing myopia earlier, and severe myopia is occurring more frequently.

Myopia is associated with genetic factors as well as socioeconomic status; however, the underlying cause for school myopia has not been established. Notably, more studies have suggested the involvement of environmental rather than genetic causes for myopia among school-aged children [2, 6]. A large body of studies have attempted to clarify the association between near work and myopia however, convincing results have not been established. Researchers believed that there must be an environmental effect that has caused the generational difference [7]. Simultaneously, recent epidemiological studies have identified outdoor activity as a key environmental determinant of myopia and revealed a protective effect of outdoor activity [8–10]. These data suggested that environmental differences may affect the onset and development of myopia.

As a country with high prevalence of myopia, China is becoming rapidly urbanized. Thus, the association between myopia and urbanization is likely to provide a better understanding of the environmental factors for myopia. Because many studies have reported a higher prevalence of myopia in urban areas compared with rural areas, living location may be important for the development and onset of myopia in children. The present study aimed to evaluate the impact of living environment on myopia among school-aged children by examining the association between housing type, parental myopia and myopia in a national sample in China.

## Methods

### Participants

The participants in this study were drawn from the “National Vision Care Related Behavior survey”, which was a cross-sectional survey conducted in 12 cities in China: Beijing, Shaoxing, Shenzhen, Chongqing, Guizhou, Taiyuan, Ma’anshan, Shenyang, Urumqi, Changsha, Yinchuan and Zhengzhou. Within each city, both rural and urban schools were included in this study; 1 elementary school and 1 junior high school were randomly chosen. A total of 43, 771 children were included in this study.

This study was conducted between March and June 2012 and was approved by the Ethics Committee of Anhui Medical University. Written informed consents of parents were obtained from all of the participants under 18 years of age for the study. The participating children or their parents were asked to complete the parental questionnaire (Additional file 1) and student questionnaire (Additional file 2), respectively.

### Assessment of variables

The questionnaires focused on personal information, parental information, living environment, time spent in physical activities, outdoors, sunlight exposure and, near workings, frequency of weekdays and weekends involved in near-work and outdoor activities. Near-work activities included doing homework, reading for pleasure, playing musical instruments, using a computer, and playing hand-held console games as well as video games and board games. Sunlight exposure time was assessed by a questionnaire inquiring about the number of hours of exposure to sunlight during a typical week day and during a typical weekend day. Participants were asked to specify the type of housing they lived in, which we classified as flat room, 1–3 floors, 4–6 floors, or 7 or more floors.

To determine whether the children and parents had myopia, we asked whether they needed to use spectacles or contact lenses and the age at which they first used them. Children and their parents were asked: *Have you been diagnosed by a medical doctor of myopia?* Participants who responded affirmatively to this question on “yes” were considered as myopia.

Physical activity was investigated by the following questions [11]: *On how many of the last 7 days did you exercise or participate in sports activities for at least 20 min that made you sweat and breathe hard, such as basketball, jogging, fast dancing, swimming laps, tennis, fast bicycling, or similar aerobic activities?; and On how many of the past 7 days did you participate in physical activity for at least 30 min that did not make you sweat or breathe hard, such as fast walking, slow bicycling, skating, pushing a lawn mower, or mopping floors?* Sufficient physical activity was defined as vigorous physical activity at least 3 days per week or moderate physical activity at least 5 days per week, using the Youth Risk Behavior Surveillance Survey (YRBSS) criteria [12].

The ethnicity categories in the present study were Han ethnicity and ethnic minority.

### Statistical analyses

Statistical analyses were performed using SPSS version 13.0 (Statistics Package for Social Science). Continuous variable was presented as mean ± *SD*, categorical variables were presented as *n* (percentage). The chi-square was performed to assess differences in the characteristic for the categorical variables. Logistic regression was used to identify the independent risk factors for myopia. The logistic regression model was performed to explore the association between the children’s myopia and parental myopia and housing type. *P* < 0.05 was considered to be statistically significant. Odds ratios (*OR*) and 95 % confidence intervals (95 % *CI*) were calculated for risk factors that were independently associated with myopia in this population.

## Results

The majority of the 43 771 children in this sample were of Han ethnicity (86.7 %, *n* = 37 951). According to the self-reported questionnaire, the prevalence of myopia among children in mainland China was 29.4 % in boys and 34.3 % in girls. Table 1 presents the demographic characteristics of the participants. The prevalence of myopia was higher in girls compared to boys, in children of Han ethnicity compared to children of other ethnicity, in those living in an urban area compared to those living in a rural area, and in children whose parents achieved a higher education level compared to those with parents of a lower education level. The mean age was 11.45 ± 2.65 years in our study participants.

**Table 1:** Distribution of myopia in children in mainland China

Characteristic	*n* (%)	Myopia		*P* value
		*n*	%	
Age (Mean ± SD)	11.45 ± 2.65			
Sex				0.000
Boys	22 225 (50.8)	6542	29.4	
Girls	21 546 (49.2)	7386	34.3	
Ethnicity				0.000
Han-ethnicity	37 951 (86.7)	12662	33.4	
Others	5820 (13.3)	1266	21.8	
Living place				0.000
Rural area	21 964 (50.2)	5641	25.7	
Urban area	21 807 (49.8)	8287	38.0	
Father’s education				0.000
Primary school and lower	6644 (15.2)	1742	26.2	
Junior high school	16832 (38.5)	4979	29.6	
Senior high school	10478 (23.9)	3612	34.5	
Some college and higher	9817 (22.4)	3595	36.6	
Mother’s education				0.000
Primary school and lower	9109 (20.8)	2452	26.9	
Junior high school	16141 (36.9)	4940	30.6	
Senior high school	9648 (22.0)	3385	35.1	
Some college and higher	8873 (20.3)	3151	35.5	

Table 2 shows the prevalence of myopia in subgroups stratified by parental myopia, housing type, near work time, sunlight exposure time, and physical activity. Compared to subjects with no parental myopia, those with one or two parents with myopia had a two and three times higher odds of self-reported myopia (*OR*: 2.09, 95 % *CI*: 1.99–2.19 and *OR*: 2.91, 95 % *CI*: 2.70–3.14, respectively). Compared to subjects living in flat room, those living in 1–3 floor, 4–6 floor and 7 floor or more floor had a higher odds of self-reported myopia (*OR*: 1.28, 95 % *CI*: 2.57–3.15, *OR*: 1.84, 95 % *CI*: 1.73–1.95 and *OR*: 2.02, 95 % *CI*: 1.88–2.16, respectively). Compared to subjects with near work time ≤ 2 h/day, those with > 2 h/day had a higher odds of self-reported myopia (*OR*: 1.38, 95 % *CI*: 1.31–1.46). Compared to subjects with sunlight exposure time ≤2 h/day, those with >2 h/day had a lower odds of self-reported myopia (*OR*: 0.90, 95 % *CI*: 0.86–0.94). Compared to subjects with no sufficient physical activity, those with sufficient physical activity had lower odds of self-reported myopia (*OR*: 0.67, 95 % *CI*: 0.63–0.71).

**Table 2:** Risk factors of myopia: Results of binary and multi variable logistic regression analysis

Variable	*n* (%)	Myopia	
		Crude *OR* (95 % CI)	Adjusted ^a^ *OR* (95 % CI)
Parental myopia			
None	8067 (26.3)	Ref.	Ref.
One	4279 (42.7)	2.09 (1.99–2.19) **	2.35 (2.22–2.49) **
Both	1582 (51.0)	2.91 (2.70–3.14) **	3.57 (3.26–3.90) **
Housing type			
Flat room	2421 (24.3)	Ref.	Ref.
1–3 floor	4440 (29.2)	1.28 (2.57–3.15) **	1.19 (1.11–1.27) **
4–6 floor	4486 (37.2)	1.84 (1.73–1.95) **	1.34 (1.25–1.44) **
7 floor or more	2581 (39.4)	2.02 (1.88–2.16) **	1.30 (1.20–1.42) **
Near work time (hours/day)			
≤ 2 h	2354 (26.4)	Ref.	Ref.
>2 h	11574 (33.2)	1.38 (1.31–1.46) **	0.96 (0.90–1.01)
Sunlight exposure time (hours/day)			
≤ 2 h	6240 (33.1)	Ref.	Ref.
>2 h	7688 (30.8)	0.90 (0.86–0.94)**	0.91 (0.87–0.96)**
Sufficient physical activity			
no	6062 (32.9)	Ref.	Ref.
yes	7866 (31.0)	0.67 (0.63–0.71) **	0.98 (0.98–0.99) **

**P<0.001

^a^Adjusted for area, gender, age, living place and ethnicity

The effect of housing type on myopia stratified by parental myopia was examined (Table 3). Table 3 presents the crude and adjusted *OR* (95 % *CI*) for each housing type group compared with the reference group (flat room) for myopia in children. An increasing prevalence of myopia was found in those children living in higher floors and in those with an increased number of myopic parents. There was a positive association between housing type and myopia with no parental myopia, one myopic parent (Table 3). After adjusting for area, gender, age, living place, ethnicity and parental education level, housing type was independently associated with myopia in both no parental myopia and one myopic parent groups. Our study suggested that housing type was not significantly related to myopia with two myopic parents (*P* > 0.001 for all).

**Table 3:** The effect of housing type on myopia among school-aged children, strastified by parental myopia

Housing type	No parental myopia			One myopic parent			Two myopic parent		
	% ^a^	Crude *OR* (95 % CI)	Adjusted ^b^ *OR* (95 % CI)	% ^a^	Crude *OR* (95 % CI)	Adjusted ^b^ *OR* (95 % CI)	% ^a^	Crude *OR* (95 % CI)	Adjusted ^b^ *OR* (95 % CI)
Flat room	20.8	Ref.	Ref.	41.4	Ref.	Ref.	47.2	Ref.	Ref.
1–3 floor	24.6	1.24 (1.16–1.34) **	1.24 (1.15–1.34) **	41.0	0.98 (0.87–1.11)	1.15 (1.00–1.32) *	44.8	0.91 (0.67–1.23)	0.99 (0.70–1.41)
4–6 floor	30.5	1.68 (1.56–1.81) **	1.56 (1.43–1.70) **	47.0	1.26 (1.12–1.41) **	1.28 (1.12–1.47) **	54.3	1.33 (0.99–1.78)	1.25 (0.88–1.78)
7 floor or more	31.0	1.72 (1.56–1.88) **	1.53 (1.37–1.70) **	47.2	1.27 (1.12–1.43) **	1.24 (1.02–1.44) **	52.9	1.26 (0.93–1.69)	1.14 (0.80–1.64)

^a^ % refers to percent of myopia in children

^b^ Adjusted for area, gender, age, living place, ethnicity and parental education level, near work, sunshine exposure and physical activity

*P<0.05, **P<0.001

**Fig. 1:**
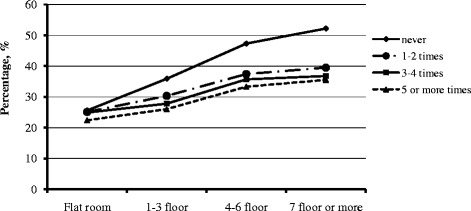
Percentage of myopia in each category of housing type and frequency of outdoor time >1 h/day in the previous 7 days (school days) among children

**Fig. 2:**
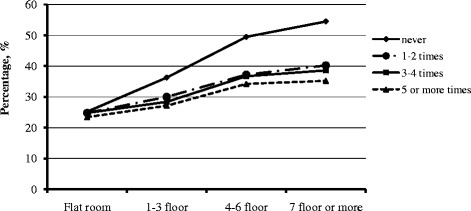
Percentage of myopia in each category of housing type and frequency of outdoor time >1 h/day in the previous 7 days (weekends) among children

Moreover, in school days, we found an increasing prevalence of myopia with increasing floor of housing type in each outdoor time group (Fig. 1). In each housing type group, the myopia rate was decreased with the increasing frequency of outdoor time, e.g., >1 h/day in the last 7 days. Similar trends were found in weekend activities (Fig. 2).

## Discussion

Myopia has rapidly increased in the last three decades [13], and is predicted to affect 2.5 billion people by the year 2020 [14]. These trends are not restricted to adults because the prevalence of myopia is also increasing in school age children [4, 13], and has emerged globally as a major public health concern [1]. In the present population-based study, the prevalence of myopia was 25.7 % in urban areas and 38.0 % in rural areas. Particularly, the findings suggested that housing type was independently associated with myopia, after stratified by parental myopia.

Our result suggested a progressive increase in the prevalence of myopia with an increasing number of myopic parents. This result are largely in line with previous studies, myopia among school-aged children has very strong family relevance, having myopic parents can increase the risk for developing myopia in children [15], and the risk may increase with the increased number of myopic parents [16]. Family relevance was considered, to a great extent, to be a hereditary factor for myopia, rather than attributed to the inheritance of myopia because family members share the same environment [17], i.e., more near-work activities and less outdoor activities. Specifically, Wojciechowski et al. [18] indicated that the recent change in the incidence of myopia is not the result of short-term shifts in genetics; instead, the secular trends in environmental and behavioral factors are perceived to be driving the myopia “epidemic”. In this regard, parental myopia is often considered as an established environmental risk factor in childhood myopia. Migrant studies may help to explore the effects of environmental exposures from genetics and thus provide additional clues to better understand the role of environmental effects on myopia [19, 20].

Although many studies have reported a higher prevalence of myopia in urban areas than in rural areas [21, 22], few studies have investigated the effect of residential housing on myopia. The present study found that housing type was independently associated with myopia, after stratified by parental myopia. Such association was also found in an Australian study, which indicates that housing type was associated with myopia among children [23]. Up to date, the effect of gene-environment interaction on the etiology of myopia is controversial because the inconsistent findings [24]. However, major changes in the prevalence of myopia among school-aged children are assumed to be associated with changes in environmental exposures, such as living areas and time spend in outdoors [6, 25]. Myopia was noted to occur significantly more frequently in children who lived in smaller, confined housing types, such as terrace houses and apartments than those who lived in standalone or separate houses [23]. Similarly, He et al. found that school locality was significantly associated with myopia [22].

Moreover, our study found an increasing prevalence of myopia with increasing floor in housing type by different frequency of outdoor activity. Ample studies have confirmed that outdoor activities are a protective factor for myopia [26, 27]. Rose et al. [28] found that a lower prevalence of myopia in Chinese children raised in Sydney compared with Chinese children living in Singapore was associated with increased hours of outdoor activities. Guggenheim et al. [29] found that both less time spent outdoors and less physical activity were associated with incident myopia and that time outdoors was found to have the larger effect. These findings were consistent with our results. Several studies hypothesized that the protective effect seems to be associated with exposure to sunshine [2, 9, 28], which was also in line with our study. Similarly, animal experiments showed the most consistent mechanism underlying the association between time outdoors and myopia is the light levels associated with outdoors [30]. Rose et al. [28] assumed that myopia might be protected by increasing the intensity of outdoor activities because of the increased release of the retinal transmitter dopamine. In an experimental study, dopamine has been found to reduce eye growth [31]. Whereas bright light was replicated by UV-free light, the protective effects were blocked by the dopamine antagonist spiperone in animals as well as in primate experiments [32].

There are two important limitations in this study. First, the estimates of myopia in children and parents were based on self-report questionnaires. In China, every year school-aged children has the routine vision screening using Standard Logarithmic Visual acuity Chart, low vision can be decided in this screening test and recorded in medical records. However, this is not the actual situation for myopia. Because for lower grade students such as grade 1 and grade 2, a large portion of low vision students are hyperopia. In order to investigate the real situation for myopia, we use the self-reported method in the national survey. However, according to the filling quality and inclusion criteria for questionnaire, we have reason for these participants provided the high quality and validity of the self-reported myopia. As a Germany study [33] showed that the difference between the self-reported refraction and the refractive error reported by their opticians was very small and not significant (*P* = 0.850), which provide evidence that self-reported refraction is reliable. Second, in our study, myopia was not measured directly among children; hence, the severity of myopia cannot be determined; the detailed refraction and ocular biometry assessments should be performed in future studies. Despite these limitations, this study is the first to report the prevalence of myopia among school-aged children and potential environmental risk factors in 12 cities in mainland China.

## Conclusions

In summary, the current study suggested a progressive increase in the prevalence of myopia with an increasing number of myopic parents. Particularly, the findings suggested that housing type was independently associated with myopia, after stratified by parental myopia. Flat room, lower living floor and more outdoor time may be protective factors for myopia among school-aged children in mainland China. These data point to the need for additional research to confirm these associations. Delineating the relationships between myopia and living environment is important for the prevention of myopia, school health workers and parents should encourage school-aged children go out for more outdoor activities.

## Additional files

Additional file 1: The National Eye Care Study Questionnaire (Parental Questionnaire for Grade 1–3 Children). (DOC 67 kb) Additional file 2: The National Eye Care Study Questionnaire (Student Questionnaire for Grade 4–9 Children). (DOC 67 kb)

## Abbreviations

95 % CI: 95 % confidence intervals
OR: Odds ratios
SD: Standard deviation
SPSS: Statistics Package for Social Science
YRBSS: Youth Risk Behavior Surveillance Survey
