# Supplementary material for: Housing type and myopia: the mediating role of parental myopia
Source: BMC Ophthalmol. 2016 Aug 31;16(1):151. doi: 10.1186/s12886-016-0324-z (PMC5006500; doi:10.1186/s12886-016-0324-z)
Supplement: Additional file 1: — The National Eye Care Study Questionnaire (Parental Questionnaire for Grade 1–3 Children). (DOC 67 kb) [file 12886_2016_324_MOESM1_ESM.doc]

**The National Eye Care Study Questionnaire**

**(Parental Questionnaire for Grade 1-3 Children)**

**Part One**

| **Gender** ① boy； ② girl | **Nationality**  ①Han nationality ②Others |
| --- | --- |
| **School** | **Grade** ①Grade 1 ②Grade 2 ③Grade 3 |
| **Registered residence**  ①rural； ②urban | **Birth date** |
| **Signature**  ①Father ②mother ③Others | **Filling Date** |

**Part Two**

**1. What is the type of houing your child living in?**

① flat room ② 1 -3 floors ③ 4-6 floors 4 ④ 7 or more floors

**2.** **Have your child been diagnosed by a medical doctor of myopia?** ① yes； ② no

**3. Father’s date of birth**

**4. Mother’s date of birth**

**5.** **How much education does your father have?**

① Primary school or less； ② Junior middle school；

③ Senior middle school； ④ College or above

**6.** **How much education does your mother have?**

① Primary school or less； ② Junior middle school；

③ Senior middle school； ④ College or above

**7. Does your biological father have myopia?** ① yes； ② no

**8. Does your biological mother have myopia?**  ① yes； ② no

Part Three Near work time

| the amount of time spent in learning (reading or writing) on a weekday | min per day in average |
| --- | --- |
| the amount of time spent in watching TV on a weekday | min per day in average |
| the amount of time spent in computer using on a weekday | min per day in average |
| the amount of time spent in mobile phone using on a weekday | min per day in average |
| the amount of time spent in learning (reading or writing) on a weekend day | min per day in average |
| the amount of time spent in watching TV on a weekend day | min per day in average |
| the amount of time spent in computer using on a weekend day | min per day in average |
| the amount of time spent in mobile phone using on a weekend day | min per day in average |

**Part Four Physial activities**

**1. Please recall or ask your child carefully, “On how many of the last 7 days did you exercise or participate in sports activities for at least 20 min that made you sweat and breathe hard, such as basketball, jogging, fast dancing, swimming laps, tennis, fast bicycling, or similar aerobic activities?”**

① 0 dyas； ② 1 dya； ③ 2 dyas； ④ 3 dyas； ⑤ 4 dyas； ⑥ 5 dyas

**2. Please recall or ask your child carefully, “On how many of the past 7 days did you participate in physical activity for at least 30 min that did not make you sweat or breathe hard, such as fast walking, slow bicycling, skating, pushing a lawn mower, or mopping floors?”**

① 0 dyas； ② 1 dya； ③ 2 dyas； ④ 3 dyas； ⑤ 4 dyas； ⑥ 5 dyas

**Part Five Outdoor activities and sunshine exposures**

**1. On sunny days, the average time of sunshine exposures (e.g. wakling, excersise, outdoor activities etc.) for your child** **on a weekday (From Monday to Friday) is about hours.**

**2. On sunny days, the average time of sunshine exposures (e.g. wakling, excersise, outdoor activities etc.) for your child on a weekend day is about hours.**

**3. On a weekday (From Monday to Friday), your child spend at least 1 hour per day on outdoor activities:**

①Never； ②1～2 times； ③3～4 times； ④ 5 or more times

**4. On a weekend day, your child spend at least 1 hour per day on outdoor activities:**

①Never； ②1～2 times； ③3～4 times； ④ 5 or more times

**小学生视力保健行为调查问卷**

**（1～3年级父母问卷）**

**一、学生情况**

| **您孩子的性别** ①男 ②女 | **您孩子的民族** ①汉族 ②其他 |
| --- | --- |
| **学校** | **年级** ①一年级 ②二年级 ③三年级 |
| **家庭所在地** ①农村 ②城市 | **孩子出生日期（阳历）** 年 月 日 |
| **填表人** ①父亲 ②母亲 ③其他 | **填表日期**  年 月 日 |

**二、家庭住房和近视情况**

**1. 您孩子的家庭住房类型：**

①瓦房或平房 ②1～3层楼房 ③4～6层楼房 ④7层以上楼房

**2. 您孩子是否曾被医生诊断为近视** ①是 ②否

**3. 生父出生日期（阳历）** 年 月 日;

**4. 生母出生日期（阳历）** 年 月 日;

**5. 生父学历** ①小学及以下 ②初中 ③高中 ④大专及以上

**6. 生母学历** ①小学及以下 ②初中 ③高中 ④大专及以上

**7. 生父是否近视** ①是 ②否

**8. 生母是否近视**  ①是 ②否

**三、请您耐心回忆在最近7天里，您孩子平均每天用眼情况。**

| 上学日（周一至周五）读书或写字时间 | 平均每天 分钟 |
| --- | --- |
| 上学日（周一至周五）看电视时间 | 平均每天 分钟 |
| 上学日（周一至周五）使用电脑时间 | 平均每天 分钟 |
| 上学日（周一至周五）玩手机时间 | 平均每天 分钟 |
| 周末读书或写字时间 | 平均每天 分钟 |
| 周末看电视时间 | 平均每天 分钟 |
| 周末使用电脑时间 | 平均每天 分钟 |
| 周末玩手机时间 | 平均每天 分钟 |

**四、体育活动**

**1. 请仔细回忆或询问您的孩子，最近7天里，有几天参与大强度的体育活动？**

（每次持续20分钟以上，出汗明显、气喘吁吁，如跑步、打球、快速骑自行车、快速游泳等）

① 0天； ② 1天； ③ 2天； ④ 3天； ⑤ 4天； ⑥ 5天及以上

**2. 请仔细回忆或询问您的孩子，最近7天里，有几天参与中等强度的体育活动？**

（每次持续30分钟以上，心跳加快，但无大量出汗或气喘吁吁，如快走、慢速骑自行车等）

① 0天； ② 1天； ③ 2天； ④ 3天； ⑤ 4天； ⑥ 5天及以上

**五、户外活动和阳光接触情况**

**1. 周一至周五，在晴天孩子通常每天平均有 小时接触到太阳（如走路、锻炼、户外活动等）。**

**2. 周末，在晴天孩子通常每天平均有 小时接触到太阳（如走路、锻炼、户外活动等）。**

**3. 您的孩子周一到周五每天户外活动大于1小时：**

① 完全没有； ② 1～2次； ③3～4次； ④ 5次以上

**4. 你的孩子周末每天户外活动大于1小时：**

① 完全没有； ② 1～2次； ③3～4次； ④ 5次以上
